# The Effect of High-Temperature Deformation on the Mechanical Properties and Corrosion Resistance of the 2024 Aluminum Alloy Joint after Friction Stir Welding

**DOI:** 10.3390/ma17122969

**Published:** 2024-06-17

**Authors:** Qiu Pang, Man Zhao, Zhichao Zhang

**Affiliations:** 1 Department of Mechanical and Electrical Engineering, Wuhan Donghu University, Wuhan 430212, China; 2 Hubei Longzhong Laboratory, Wuhan University of Technology, Xiangyang 441000, China; manzhao0112@163.com; 3 Aerospace Equipments Manufacturer Co., Ltd., Shanghai 200245, China; chao2010@yahoo.com

**Keywords:** 2024 aluminum alloy, friction-stir-welded joint, high-temperature deformation, corrosion resistance

## Abstract

The 2024 aluminum alloy is one of the high-quality lightweight materials. Friction stir welding (FSW) has shown advantages in reducing welding defects and improving welding quality in 2024 aluminum alloys. Currently, the research regarding FSW joint corrosion performance is mainly about the joint without plastic deformation. However, FSW joints often need to be formed into complex shapes by plastic deformation. The influence of plastic deformation on the corrosion performance of FSW joints is the focus of scientific research. To address this problem, the effect of high-temperature deformation on the mechanical properties and corrosion behavior of 2024 aluminum alloy joints was researched. The exfoliation corrosion test, scanning electron microscopy, energy-dispersive spectroscopy, and transmission electron microscopy were employed to analyze the corrosion mechanism and microstructure. The results show that high-temperature deformation of the weld nugget zone greatly affects the mechanical properties and corrosion behavior of the FSW joint. Compared with the 0% deformation specimen, the hardness and tensile strength of the 20% deformation FSW joint increased by 32% and 21%, respectively. The FSW joint with 20% deformation shows the best mechanical properties and corrosion resistance. The number of precipitated S’ phases of the FSW joint increases when the deformation increases to 20%, and the shape of the S’ phase is a regular round particle shape. The dislocation density of the FSW joint increases continuously during deformation, which provides a favorable nucleation location for the S’ phase.

## 1. Introduction

With the rapid development of aerospace and high-end equipment, the high performance and light weight of formed parts have attracted much attention from scholars, so lightweight and high-strength materials have become the focus of research [1,2]. Energy saving, emission reduction, and improved safety have become some of the important directions of contemporary industrial development. Ensuring a product is lightweight is one of the important ways to achieve energy savings and emission reductions and improve product performance. Compared with other lightweight materials, high-strength aluminum alloys are special lightweight materials because of their unique advantages [3,4,5]. Specifically, 2024 aluminum alloy, with its good formability and serviceability, is widely used in the aerospace field, being used to construct products such as skeleton parts and skin and other complex components on aircraft [6,7]. However, for such large, thin-walled complex parts, a single piece of material is not used. Instead, several metal plates of different materials and different sizes are joined together to form complex shapes. The application reliability can be greatly improved by adopting tailor welded blank forming [8]. However, when traditional fusion welding is used to weld 2xxx aluminum alloy, welding defects such as pores or thermal cracks occur, and the mechanical properties of the joint are reduced [9]. 

Friction stir welding (FSW) is an advanced manufacturing technique employed to achieve the light weight of high-end equipment. At the same time, it is also the forefront of the development of the international forming manufacturing field. At present, FSW technology overcomes the defects of traditional welding, being without welding wire and inert shielding gas, which means it can effectively refine grain size and homogenize the microstructure [10]. Moreover, FSW has a less drastic impact on the environment due to solid-state green welding, which does not involve any fumes, red-hot glowing materials, spattering, or loud noise during operation [11]. FSW provides the perfect technical solution for welding aluminum [12], magnesium [13], copper [14], titanium [15], steel [16], and other metal materials [17], producing low-cost and high-quality joints. Therefore, it could become widely used in modern industries for joining either homogeneous or dissimilar materials. There are various engineering applications, such as aviation thin-wall structural parts, aerospace cylindrical structural parts, high-speed train body structures, and so on. Ahmed [18], in 2015, successfully welded 0.44 mm 6xxx Al sheets with butt and lap joint configurations. The results proved that the transverse tensile strength was better than the longitudinal strength. Yue et al. [19] found that a 0.08 mm shoulder plunge depth was sufficient for reducing sheet thickness. A maximum tensile strength of 399.5 MPa and an elongation of 5.6% were achieved at 1000 rpm and 150 mm/min.

Because high-end aerospace equipment is exposed to a variety of different environments, such as high-humidity and high-salt environments, during service, intergranular corrosion and spalling corrosion can easily occur, which directly affects the performance and lifespan of the equipment. In the past decade, extensive research has been carried out on the corrosion behavior of aluminum alloy FSW joints [5,20,21,22,23]. For example, Thamilarasan et al. researched the salt fog corrosion behavior of friction-stir-welded AA2014-T651 aluminum alloy. The results showed that the corrosion resistance of the welds and parent material in the basic solution was better than that in acidic and neutral solutions [24]. Qin et al. investigated the corrosion behavior of friction-stir-welded 2A14-T6 aluminum alloy in immersion exfoliation corrosion (EXCO) solution. The results obtained showed that, compared to the base material, the corrosion resistance of the friction-stir-derived welds was greatly improved and the weld nugget had the highest corrosion resistance [25]. 

However, the research results of traditional panels cannot accurately reflect the influence of deformation on the corrosion resistance of FSW joints because the FSW-welded aluminum alloys need to be formed into complex shapes after plastic deformation. An elongated grain structure is an important requirement for exfoliation corrosion and material machined from the center of a wrought plate [26,27,28]. At present, the studies of deformation’s influence on the corrosion properties of aluminum alloys mainly focus on the influence of pre-deformation and heat treatment processes on the mechanical properties and corrosion properties of aluminum alloy base metal [29,30]. Hu et al. [31] investigated the microstructural evolution and mechanical properties of friction-stir-welded joints during plastic forming. The results obtained showed that the tensile and yield strengths of the friction-stir-welded joints were significantly improved after severe plastic deformation due to the grain refinement. However, only a few studies have focused on the mechanical properties and corrosion resistance of FSW joints under high-temperature deformation.

In this study, the effect of high-temperature deformation on the mechanical properties and corrosion behavior of 2024 aluminum alloy joints were researched. The corrosion microstructure and mechanism were analyzed by electrochemical measurements, scanning electron microscopy, and transmission electron microscopy. Relationships among deformation amount, corrosion property, and microstructure evolution are established. A technical method for ensuring the high corrosion resistance of 2024 aluminum alloy FSW joints is developed. It provides a theoretical basis and technical support for improving the corrosion resistance of high-end aviation equipment.

## 2. Materials and Methods

### 2.1. Material and Sample Preparation

**Figure 1:**
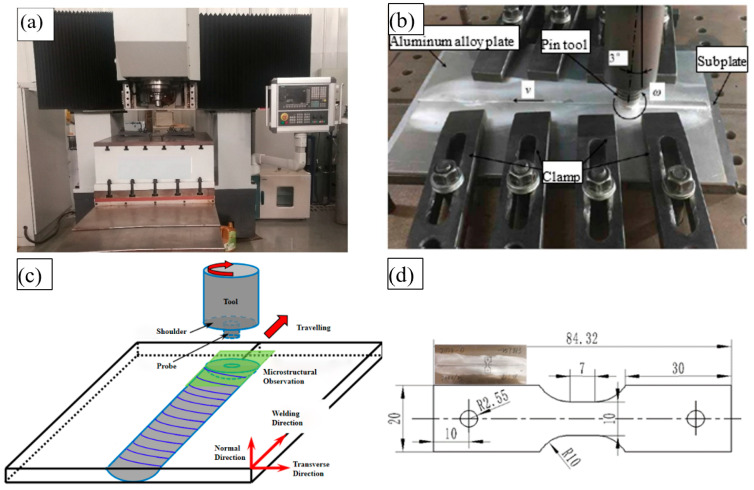
(**a**,**b**) A photograph of friction stir welding equipment; (**c**) the 3D model of the sample preparation of friction stir welding; (**d**) the size of the high-temperature tensile sample (units: mm).

The base metal in the test was the 2024-O aluminum alloy with a sheet thickness of 2 mm and it was obtained from Alnan Aluminum Co., Ltd. (Nanning, China). The sample size was 300 mm × 80 mm × 3 mm. The friction stir welding equipment was the NFSW-650 model developed by the Shenyang Institute of Automation of the Chinese Academy of Sciences, as shown in Figure 1a,b. When welding, a butt joint was used to join the 2024-O aluminum alloy plates. According to the research on friction stir welding with the 2024-O aluminum alloy [32], the travel speeds were 10–100 mm/min and the rotation speeds were 800–2500 r/min. In this experiment, the process parameters of FSW were a rotation speed of 2000 rpm and travel speeds of 25 mm/min and 100 mm/min. An FSW head was created with a tool made of 4Cr5MoV tool steel. The shaft shoulder diameter of the tool was 10 mm. The pin diameter was 3 mm. The pin height was 2.85 mm and the inclination angle was 3°. The lower pressure of the control shaft shoulder was 0.1 mm–0.2 mm. 

A 3D model of the sample preparation for friction stir welding is shown in Figure 1c. The welding direction was perpendicular to the rolling direction of the base metal. The size of the high-temperature tensile sample is shown in Figure 1d. According to the standard GB/T 288.1-2010 [33], the tensile samples were cut perpendicular to the weld. A high-temperature deformation experiment was carried out on an MMS-200 thermal simulation experimental machine, which was developed by Northeastern University.

**Figure 2:**
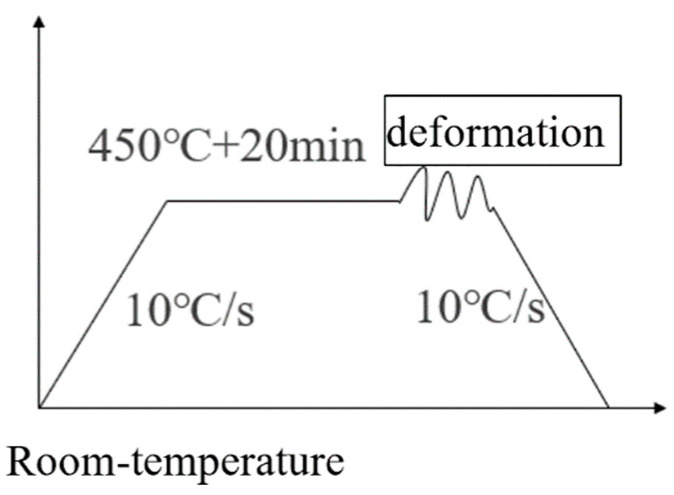
Processing flow of the high-temperature tensile sample.

At first, the thermal simulation testing machine was heated by direct resistance at a heating rate of 10 °C/s. In order to avoid the generation of abnormal grain growth (AGG), the FSW joints of the 2024-O aluminum alloy samples underwent a solution heat treatment at 450 °C for 20 min. Then, the samples were thermally stretched at a strain rate of 0.01 s^−1^, followed by immediate quenching in cold water. The high-temperature deformations were 0%, 10%, 20%, and 30%, respectively. The processing flow for the high-temperature tensile samples is presented in Figure 2. Each sample group was subjected to three tensile tests to ensure the reproducibility of the results. 

**Figure 3:**
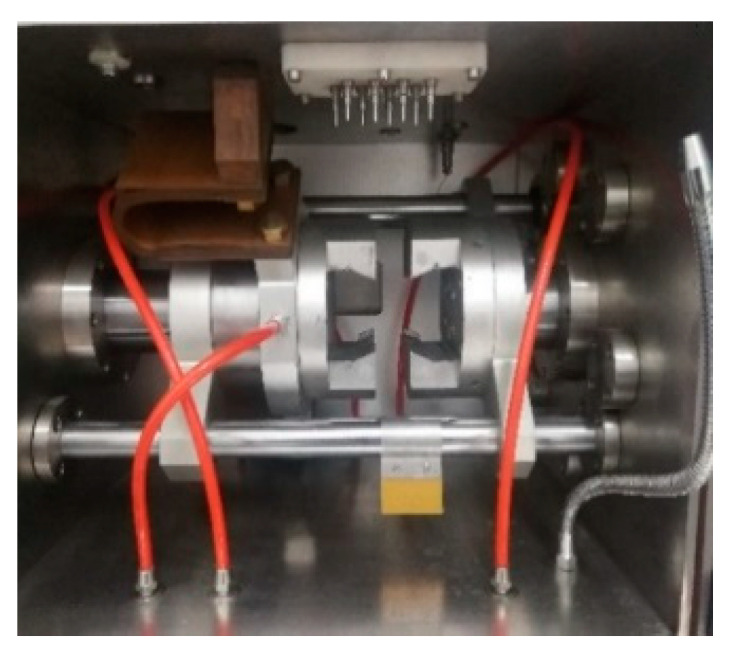
A clamp with embedded water cooling.

To provide accurate feedback and control of the temperature signal, a sample with a welded thermocouple pair was mounted on a clamp with high thermal conductivity and a clamp that was embedded in water for cooling was used, as shown in Figure 3.

### 2.2. Corrosion Experiment

Before the corrosion experiment, samples with dimensions of 10 × 10 × 1.5 mm were machined from the 2024-O aluminum alloy FSW joint and were then cleaned in an ultrasonic cleaning machine with alcohol for 10 min. Then, 400-grit, 800-grit, and 1200-grit sandpaper was used to perform rough grinding and fine grinding. Each sample was then polished with a YMPZ-2 metallographic sample polishing machine manufactured by Shanghai Metallurgical Equipment Company Ltd. (Shanghai, China). An epoxy resin was used as a filling agent for the corrosion tests and silica gel was used to seal.

An exfoliation corrosion experiment was conducted according to GB/T 22639-2008 [34]. The cutting position of the sample was the same as that in the electrochemical experiment. The ratio of the solution was 236 g (2.16 g/mL) NaCl + 50 g (2.10 g/mL) KNO_3_ + 6.3 mL (1.40 g/mL) HNO_3_; it was diluted to 1000 mL with distilled water and sealed with rosin on the nonworking surface. The samples were soaked at a temperature of 25 ± 2 °C and a humidity of 45 ± 6% for 24 h, 48 h, and 96 h and were then taken out. After the corrosion experiment, the samples were thoroughly cleaned. Subsequently, the samples were dried in a drying oven at 100 °C for 6 h. Finally, the samples were weighed using a balance.

An intergranular corrosion experiment was conducted according to GB/T 7998-2005 [35]. The cutting position of the sample was the same as that in the electrochemical test, and the size was 10 × 10 × 1.5 mm. The samples were successively placed in NaOH solution and HNO_3_ solution to remove the surface oxide layer. A corrosive solution was prepared with 57 g/L NaCl + 10 mL 0.4 mol/L H_2_O_2_. The solution temperature was between 23 °C and 27 °C and the corrosion time was 6 h.

The samples were polished and mirrored under a water trickle using different abrasive papers. The polished specimens were etched with Keller’s reagent (190 mL of distilled water, 5 mL of HNO_3_, 3 mL of HCl, and 2 mL of HF). The chemical agents were provided by Hubei Zhongshui Chemical Co., Ltd. Metallographic observation was carried out with a Zeiss Scope-A1 metallographic microscope (OM, Axiovert 200 MAT, Carl Zeiss Inc., Oberkochen, Germany). The microstructure was examined using a field-emission scanning electron microscope (FESEM, Hitachi S-4800, Tokyo, Japan). The microhardness of the high-temperature deformation sample was measured using an HV-1000A Vickers hardness tester (Junda Times Instrument Co., Ltd., Shenzhen, China) at different regions of the weld, with an applied load of 200 g and an indentation time of 10 s. The precipitate distributions in the as-welded joints were observed through transmission electron microscopy (TEM, Tecnai 20, FEI Company, Hillsboro, OR, USA).

## 3. Results and Discussion

### 3.1. Morphology of FSW Joints

**Figure 4:**
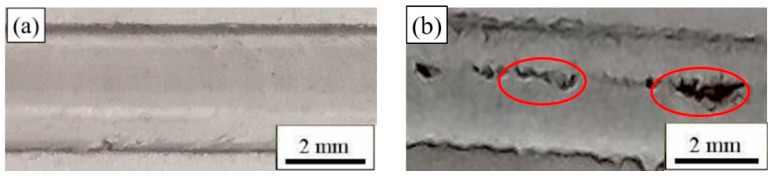
Macro-morphology of 2024-O aluminum alloy FSW joint at different tool travel speeds with a constant rotational speed of 2000 r/min: (**a**) travel speed of 25 mm/min, (**b**) travel speed of 100 mm/min.

Based on existing research [32], the welding rotational speed was set to 2000 r/min. Figure 4 shows the macro-morphology of the FSW joints at different tool travel speeds.

The travel speeds of the stirring tool had a great influence on the surface morphology of the 2024-O aluminum alloy. When the travel speed was 25 mm/min, the surface microstructure was smooth and compact. The results showed that the welding quality with FSW was excellent, as shown in Figure 4a. When the travel speed was increased to 100 mm/min, the contact time of the stirring head and sample was short and the heat input was insufficient. Micro-holes and cracks occurred in the welding process, as shown in Figure 4b [36] (as shown in the red circles). 

**Figure 5:**
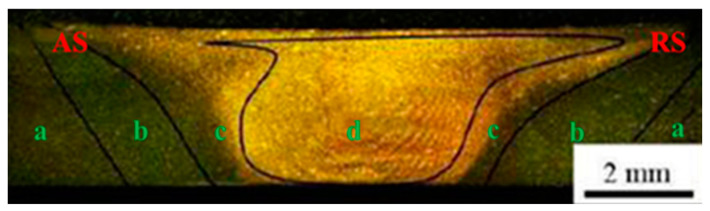
The cross-sectional macrostructures of the 2024-O aluminum alloy FSW joint: (**a**) the base metal zone, (**b**) heat-affected zone, (**c**) thermomechanically affected zone, (**d**) weld nugget zone.

Figure 5 illustrates the transverse cross-sections of the FSW joint of the 2024-O aluminum alloy at a rotational speed of 2000 r/min and a travel speed of 25 mm/min. In the figure, the left side is the advancing side (AS) of the joint and the right side is the backward side (RS) of the joint. No welding defects were detected in the FSW joints. Different regions of the FSW joints are also shown in Figure 5.

The base metal zone, heat-affected zone, thermomechanically affected zone, and weld nugget zone are represented by the letters a, b, c, and d, respectively. The distribution region was approximately symmetrical. Due to the different thermal effects of the stirring needle in the whole welding process, the degree of plastic deformation of each part in the weld was different. The weld nugget zone in the sample was basin-shaped [37]. Simultaneously, the weld nugget zone was located directly below the stirring head during the welding process, and dynamic recrystallization occurred in the weld nugget zone, which could promote the redistribution of stresses [38].

**Figure 6:**
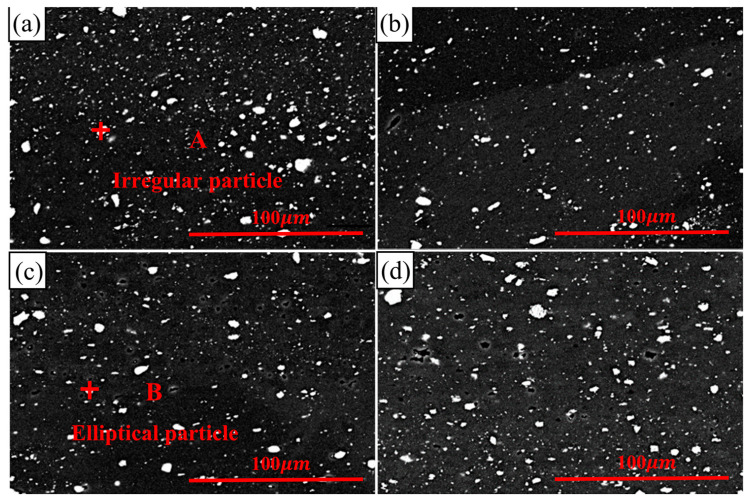
The microstructure of 2024-O aluminum alloy FSW joint with different high temperature deformations: (**a**) 0%, (**b**) 10%, (**c**) 20%, (**d**) 30%.

**Figure 7:**
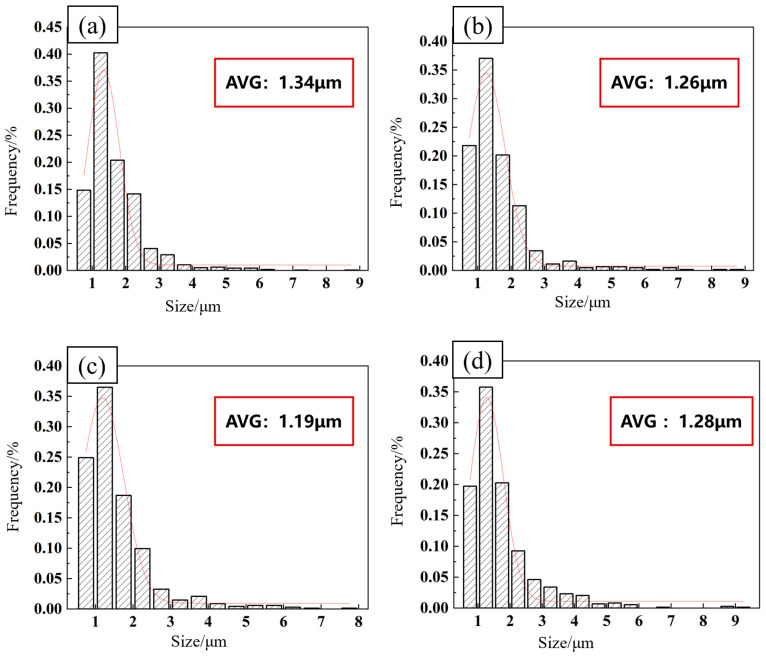
The mean value of dispersed particles by image processing: (**a**) 0%, (**b**) 10%, (**c**) 20%, (**d**) 30%.

Figure 6 shows the microstructures of the FSW joints of the 2024-O aluminum alloy after different high-temperature deformations, and the mean values of the dispersed particles obtained via image processing are shown in Figure 7.

**Figure 8:**
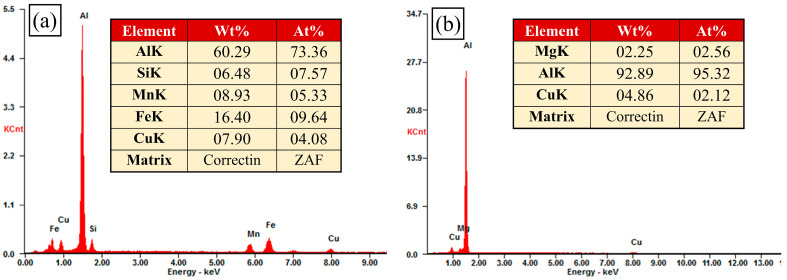
EDS spectra from different high-temperature deformations: (**a**) 0%, (**b**) 20%.

Figure 8 illustrates the EDS spectra of the two points enumerated in Figure 6.

The mechanical properties of aluminum alloy FSW joints are closely related to their microstructure. Some irregular and round-shaped particles were distributed in the matrix, with an average size of 1.34 μm for the joint without high-temperature deformation (Figure 7a and Figure 8a). The irregularly shaped particles were 8–12 times larger than the round particles. The EDS spectrum of point A showed the elements Al, Si, Mn, Fe, and Cu. The intensity of the Al peak was high and the peaks of the other elements were low, as shown in Figure 8a. The irregular and coarse particles proved to be an undissolved phase [39]. With the appearance of high-temperature deformation, the coarse particles were broken. Elliptical particle shapes were evenly distributed, with an average size of 1.26 μm; these were shown to be the S’ phase and they provided more nucleation sites for the formation of recrystallized grains, as shown in Figure 7b and Figure 8b [40]. These fine and dispersed particles were enhanced precipitated phases [41].

When the deformation increased from 10% to 20%, the second phases were more evenly distributed, as shown in Figure 6c and Figure 7c. The amount of white precipitated phases increased and the distributions were finer and more uniform, with an average size of 1.19 μm, when the level of high-temperature deformation was 20%. However, when the amount of deformation was 30%, the size of the dispersed particles increased to 1.28 μm, as shown in Figure 7d. This was because the specimen underwent more serious high-temperature deformation.

### 3.2. Mechanical Properties of the 2024-O Aluminum Alloy FSW Joint

**Figure 9:**
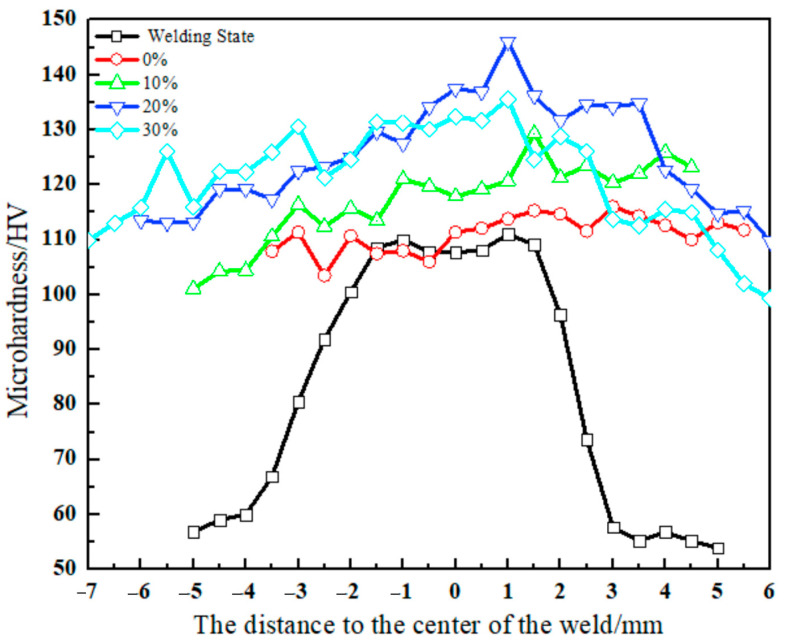
The microhardness distribution of cross-section of 2024-O aluminum alloy FSW joint with different high-temperature deformations.

Figure 9 shows the microhardness distributions in a cross-section of the 2024-O aluminum alloy FSW joints after the welding state and different high-temperature deformations.

In the welding state, the microhardness of the 2024-O aluminum alloy FSW joints showed an obvious “Ω” shape. The interval of [−1.5, 1.5] belonged to the weld nugget zone, the intervals of [−3.5, −2] and [2, 2.5] belonged to the thermomechanically affected zone, and the rest belonged to the heat-affected zone and base metal zone. The joint hardness distributions were as follows, from high to low: weld nugget zone, thermomechanically affected zone, heat-affected zone, and base metal zone [42]. The average microhardness of the base material was 65 HV and the microhardness of the heat-affected zone was basically the same as that of the base material. The average microhardness of the weld nugget zone was the highest and its value was 111 HV. The microhardness of the weld nugget zone was 1.7 times that of the base metal zone.

This was mainly because the base metal was subjected to the stirring shear action of the stirring head in the welding process. The coarse-phase particles were broken and the precipitated S’ phases were evenly distributed, which played a role in precipitation strengthening. Simultaneously, a short-term solid solution occurred in the thermomechanically affected zone under the action of welding heat, which played a role in solid solution strengthening. The further the distance from the welding core zone, the weaker the strengthening effect of the solution [43]. In general, the weld nugget zone of the specimen with 20% high-temperature deformation had the highest hardness values, and the average hardness value was 120 HV. The results showed that the average hardness value of the same material was increased by 14% compared with that in the literature when the rotation speed was 1500 rpm and the travel speed was 120 mm/min [44]. In addition, the effects of high-temperature deformation on the second-phase particle distributions led to an increase in the hardness.

**Figure 10:**
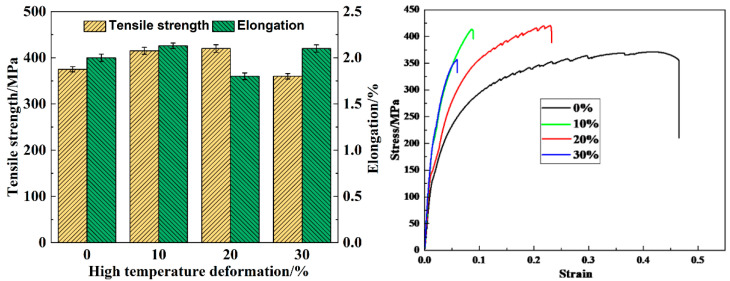
The tensile strength, elongation, and stress–strain curves at room temperature of 2024 aluminum alloy FSW joint with different high-temperature deformations.

Figure 10 shows the tensile strength, elongation, and stress–strain curves of the 2024-O aluminum alloy FSW joints at room temperature with high-temperature deformations of 0%, 10%, 20%, and 30%.

The average tensile strength of the 0% deformation sample was 375 MPa at room temperature. The average tensile strengths of the 10%, 20%, and 30% deformation samples were 415 MPa, 420 MPa, and 360 MPa, respectively. It was observed that the tensile strength of the joint increased after appropriate high-temperature deformation, which was consistent with the trend of the variations in hardness. When the high-temperature deformation was 20%, the tensile strength of the FSW joint reached the maximum value of 465 MPa. Compared with the values in the literature under the conditions of a rotation speed of 2500 rpm and welding speed of 50 mm/min for heterogeneous 7075-2024 aluminum alloys, the tensile strength of the FSW joint with 20% deformation increased by 14% [32]. This was because the deformation led to an increase in the dislocation density and formed a dislocation network in the matrix, which resulted in enhancing the strength of the pre-deformed sample [45]. Simultaneously, the increase in dislocation provided a favorable place for the nucleation of the precipitated phase, which increased the precipitation quantity and uniformity in the process of artificial aging. However, when the high-temperature deformation increased to 30%, the tensile strength of the FSW joint decreased. This was due to excessive dislocation entanglement and aggregation, forming dislocation cells, which not only provided power for precipitate growth but also caused a coarse precipitate to be formed [46]. Therefore, the tensile strength of the specimens with different degrees of high-temperature deformation was as follows from high to low: 20% > 30% > 10% > 0%. 

### 3.3. Fracture Morphology Analysis of the FSW Joint

**Figure 11:**
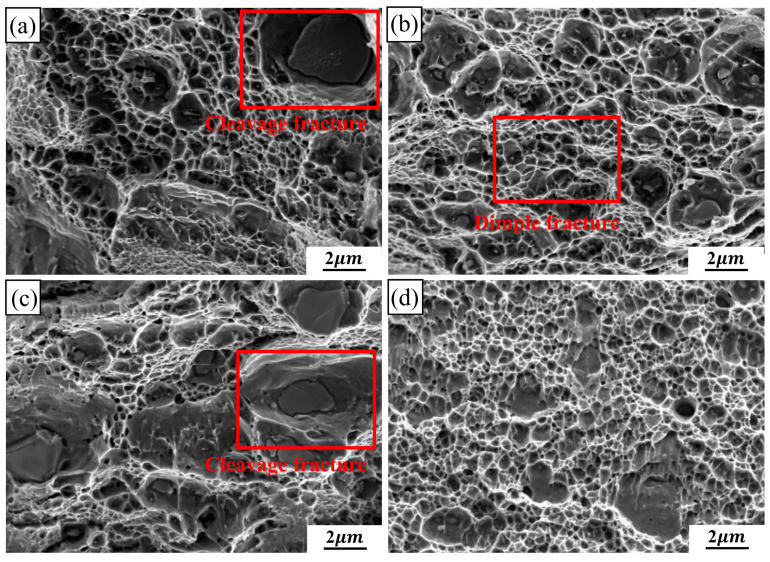
The room-temperature tensile fracture morphology of the 2024 aluminum alloy FSW joint with different high-temperature deformations: (**a**) 0%, (**b**) 10%, (**c**) 20%, (**d**) 30%.

Figure 11 shows the tensile fracture morphology at room temperature for the 2024 aluminum alloy FSW joints with different degrees of high-temperature deformation.

It can be observed that the FSW joints with 0% and 20% deformation are the cleavage and dimple fractures, and a clear cleavage plane can be observed in Figure 11a,c, as shown in the red square. Because the cleavage fracture belongs to the brittle fracture form, the fracture process is discontinuous [47]. The elongations of 0% and 20% deformation samples are less than 2% in Figure 10. However, the dimple fracture is the main fracture mode on the 10% and 30% deformation specimens in Figure 11b,d. Dimples are the main microscopic feature of metal plastic fracture. Simultaneously, due to the tearing stress, the dimples are elongated and the shapes are parabolic [48]. Compared with the joint of 0% deformation specimen, 10% and 30% deformed joints have more continuous dimples. Especially, the dimple depths increase when the deformations increase to 10%. However, the dimples of 20% deformation specimens are less on the fractures. The results are consistent with the distribution of elongation values of the specimens with different high-temperature deformation in Figure 10.

### 3.4. The Corrosion Behavior of High-Temperature Deformation Samples

**Figure 12:**
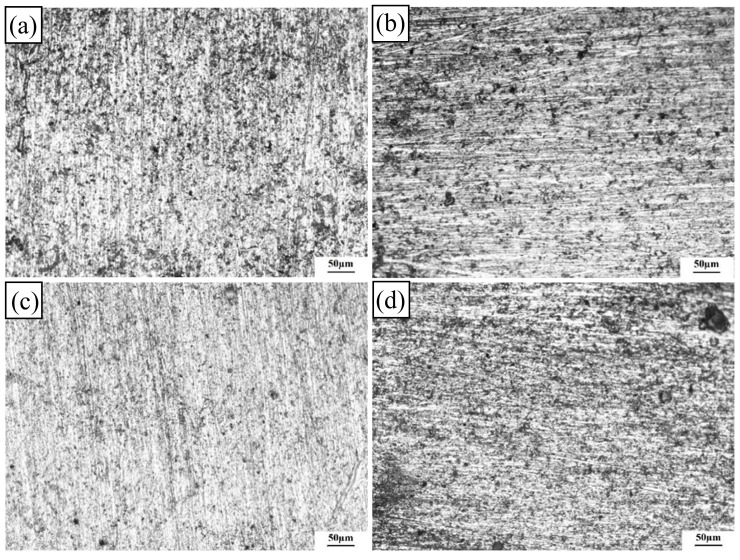
Exfoliation corrosion morphologies of the 2024 aluminum alloy FSW joints with different high-temperature deformations after 24 h: (**a**) 0%, (**b**) 10%, (**c**) 20%, (**d**) 30%.

Figure 12 shows the exfoliation corrosion morphologies of the 2024-O aluminum alloy FSW joints with different degrees of high-temperature deformation after 24 h of corrosion.

The corrosion solution was a saturated NaCl solution with a mass fraction of 3.5%. The main type of corrosion attack was pitting corrosion. When the corrosion time was 24 h, the pitting degree of the specimen surface was relatively slight. The corrosion morphologies of all samples showed dense and uniformly distributed pitting defects. However, the size and distribution of the pits varied with the increase in deformation. The 0% undeformed and 10% deformation samples showed severe corrosion, as shown in Figure 12a,b. Some pitting holes were detected. The number and size of the pits were slightly greater. Compared with the number of pits in the 10% deformation sample, there were fewer pits in the 20% deformation sample and the distribution was sparse, as shown in Figure 12c. However, the pits in the 30% deformation sample were more distributed than those in the 20% deformation sample, as shown in Figure 12d. The results showed that the FSW joint with 20% deformation had enhanced pitting resistance. This was consistent with the electrochemical results discussed above.

**Figure 13:**
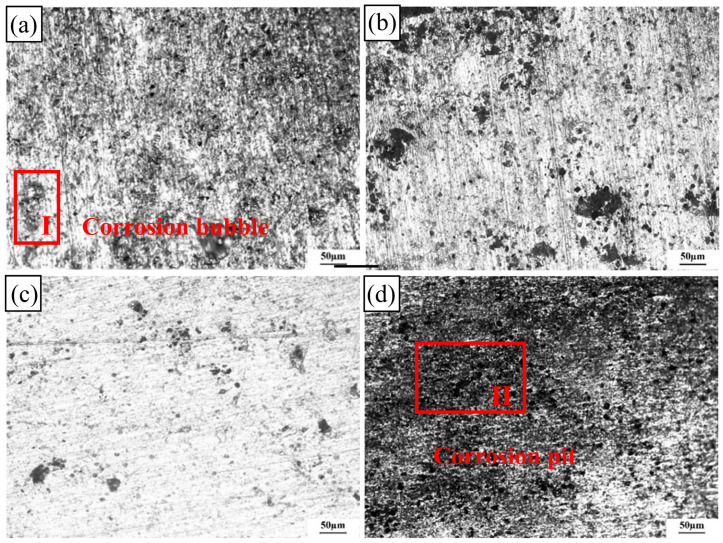
Exfoliation corrosion morphologies of the 2024 aluminum alloy FSW joints with different high-temperature deformations after 48 h of corrosion: (**a**) 0%, (**b**) 10%, (**c**) 20%, (**d**) 30%.

Figure 13 illustrates the exfoliation corrosion morphologies of the 2024-O aluminum alloy FSW joints with different degrees of high-temperature deformation after 48 h of corrosion.

The pit size of the 0% deformation sample after 48 h of corrosion was significantly larger than that of the sample after 24 h of corrosion and the distribution of pits was denser. A few pits began to expand to form pitting bubbles and the corrosion bubbles showed a large amount of black mist, as shown in Figure 13a (red zone I). Compared with that of the 0% deformation sample, the corrosion of the 10% deformation sample was more serious after 48 h of corrosion, as shown in Figure 13b. The 10% deformation sample showed larger pits and some pits slowly accumulated to form corrosion bubbles. However, the 20% deformation sample had good corrosion resistance and some areas had sparse pits, as shown in Figure 13c. When the 30% deformation sample was corroded for 48 h, intense pitting took place and pitting holes were distributed in every region. The dark-field image of the corroded surface in Figure 13d clearly exhibits a thick covering of corrosion products.

**Figure 14:**
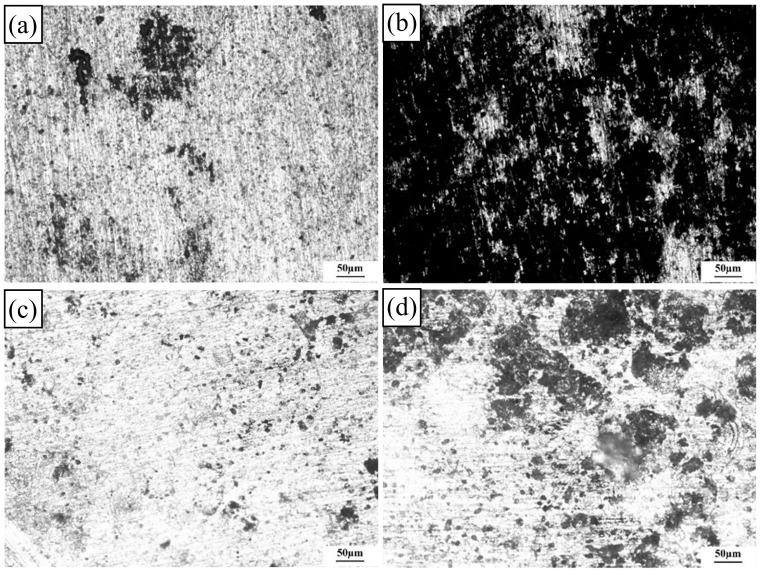
Exfoliation corrosion morphologies of the 2024 aluminum alloy FSW joints with different high-temperature deformations after 96 h of corrosion: (**a**) 0%, (**b**) 10%, (**c**) 20%, (**d**) 30%.

When the exfoliation corrosion time was 96 h, the pitting degree increased, as shown in Figure 14.

The pits of the 0% deformation specimen formed grooves. In addition, a small number of corrosion bubbles appeared on the surface and the corrosion bubbles were interconnected, eventually leading to the stripping of the corrosion products’ surface. In particular, the corrosion of the 10% deformation sample was the most serious and large corrosion pits were formed on the surface of the sample. The area and depth of the pits simultaneously increased, as shown in Figure 14b. Delamination and spalling occurred alternately, resulting in multilayer spalling. Compared with those of the 0% and 10% deformation samples, the pits of the 20% deformation sample were few and evenly distributed. The corrosion layer presented a relatively flat and smooth area. The equiaxed grain structure could greatly improve the exfoliation corrosion resistance. However, when the high-temperature deformation was 30%, some pits on the sample surface clustered together to form larger and deeper corrosion holes. Powdering or flaking of the surface occurred. This was because excessive deformation could cause some defects in the crystal and reduce the corrosion resistance of the 2024-O aluminum alloy FSW joints [49]. It can be concluded that the corrosion resistance of the specimens with different levels of deformation was as follows from high to low: 20% > 0% > 30% > 10%. This result was consistent with the electrochemical results discussed above.

**Figure 15:**
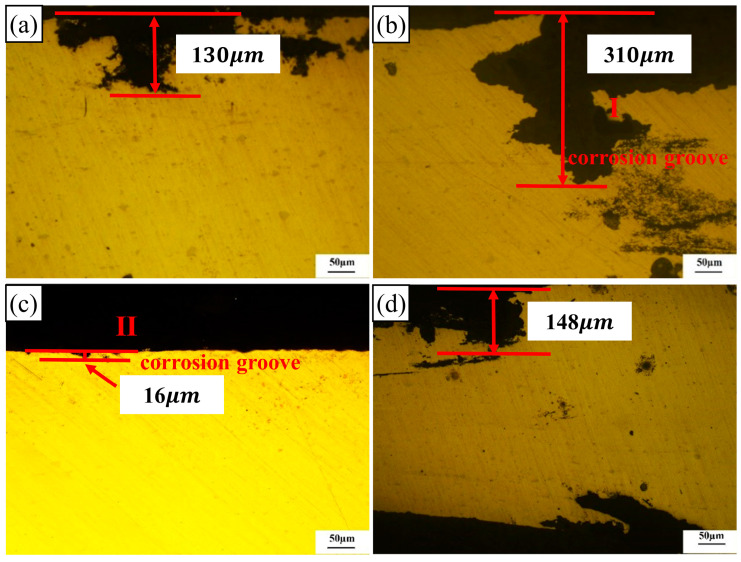
Intergranular corrosion morphologies of the 2024 aluminum alloy FSW joints with different high-temperature deformations: (**a**) 0%, (**b**) 10%, (**c**) 20%, (**d**) 30%.

Figure 15 illustrates the intergranular corrosion morphologies of the 2024-O aluminum alloy FSW joints with different degrees of high-temperature deformation.

High-temperature deformation had a great influence on the corrosion properties of the samples. Intergranular corrosion occurred in a layered structure. The cross-sections of the 0%, 10%, and 30% specimens showed serious intergranular corrosion and corrosion grooves along the rolling direction. According to the actual measurement, the maximum depth of the corrosion pit for the 0% deformation sample was 130 µm and the pits were located around the deep grooves, as shown in Figure 15a. The corrosion pit in the 10% deformation specimen exhibited the deepest level of intergranular corrosion compared with the other three samples and the maximum depth value was 310 µm, as shown in Figure 15b. However, the maximum depth value of the corrosion pits of the 20% deformation specimen was only 16 µm and the tendency of intergranular corrosion was weak, as shown in Figure 15c. The maximum depth of the corrosion pits in the 30% deformation specimen was 148 µm. Some small cracks were connected with the corrosion surface, thereby forming corrosion pits along the grain boundaries, as shown in Figure 15d. In summary, the corrosion degree of the 20% deformation specimen was the lowest, which was consistent with the previous electrochemical test.

In the corrosion process of aluminum alloy FSW joints, the fundamental cause of corrosion is the formation of electric couples between the matrix and the second phase. Because of the different phases between grains, the grain boundaries are in a state of disorder. As a result, grain boundaries have more stored energy than grain interiors. The S’ phase easily precipitates and aggregates at the grain boundary, where intergranular corrosion easily occurs. After heat treatment, the main precipitated phase of the 2024-O aluminum alloy was the S’ phase, which contained Cu [50]. When Cu is precipitated as the S’ phase in a supersaturated solid solution, corrosion mainly occurs in the copper-poor zone. The direction of corrosion expansion was consistent with the direction of the distribution of the joint-strengthening phase, which was along the rolling direction. In addition, the precipitated phase of the joint was broken and dispersed evenly in the matrix under the action of the friction stir welding stirring head. The copper-poor zone precipitated between grains. There was an electric potential difference between copper-poor and copper-rich regions, which led to the formation of corrosion microcells and accelerated corrosion. Intergranular corrosion occurred and spread along grain boundaries. As the corrosion time increased, the morphology of intergranular corrosion cracks was formed.

**Figure 16:**
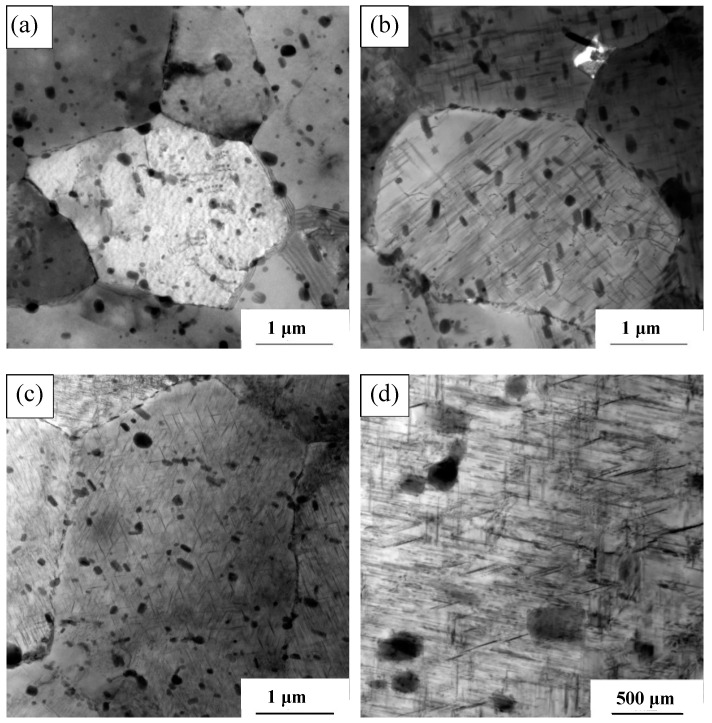
The precipitated phase morphology of the 2024 aluminum alloy FSW joints with different high-temperature deformations: (**a**) 0%, (**b**) 10%, (**c**) 20%, (**d**) 30%.

Figure 16 shows the TEM morphology of the 2024-O aluminum alloy FSW joints with different degrees of high-temperature deformation (0%, 10%, 20%, and 30%).

Figure 16a illustrates the 0% deformation sample that only underwent solid solution and artificial aging heat treatment. It can be seen in the figure that the number of precipitates in the 0% deformation sample was relatively low, and the distribution was sparse and uneven. The matrix underwent significant changes as the deformation increased, and the number of precipitates increased. When the deformation amount was 10%, the size of the precipitated phase was generally coarse, as shown in Figure 16b. Simultaneously, some precipitated phases were relatively sparse and the corresponding size was large.

When the deformation amount increased to 20%, as shown in Figure 16c, the size of the precipitated phase significantly decreased and the amount of the precipitated phase increased. The overall distribution was more uniform, and the shape of the precipitated phase was a very fine needle structure. This was primarily due to the continuous increase in dislocation density, which provided a more favorable site for the precipitation of the S’ phase and, consequently, more of the S’ phase began to nucleate [51]. However, when the deformation increased to 30%, as shown in Figure 16d, the size of the precipitated phase was larger than that in the 20% deformation sample and its distribution was not as uniform as that in the 20% deformation sample. The dislocation density of the 30% deformation sample further increased, which provided an impetus for the growth of the S’ phase, leading to the coarsening of the S’ phase.

**Figure 17:**
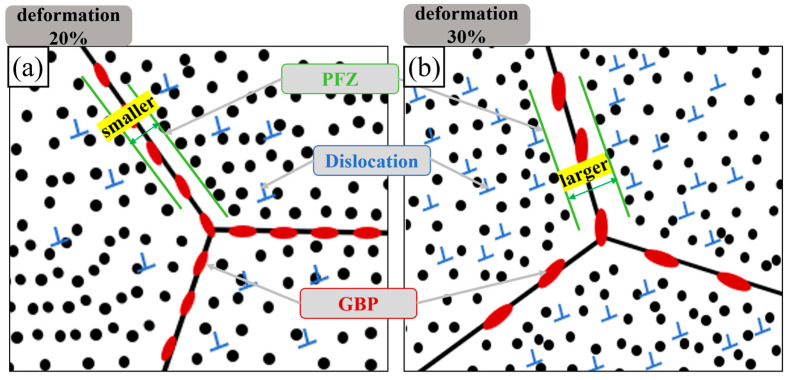
The precipitated phase morphology of the 2024 aluminum alloy FSW joints with high-temperature deformations: (**a**) deformation 20%, (**b**) deformation 30%.

Based on the analysis of the results above, a schematic diagram of the precipitated phase morphology with high-temperature deformation was drawn, as shown in Figure 17.

The 20% deformation sample exhibited the greatest amount and density of S’ phase precipitation, with a large amount of uniformly distributed precipitates. Cu and Mg atoms near the grain boundary were absorbed and the non-precipitation zone was further depleted of Cu and Mg. The grain boundary precipitates were reduced in quantity and their distribution was discontinuous. The non-precipitation zone became narrower, as shown in Figure 17a, significantly reducing the likelihood of corrosion and improving corrosion resistance. In comparison with the 20% deformation sample, the 30% deformation sample exhibited less of a reduction in S’ phase precipitation at the grain boundary due to the coarser and more uneven distribution of precipitates, with a relatively wider non-precipitation zone, resulting in less favorable corrosion resistance [52]. Conversely, the 10% deformation sample generated the fewest dislocations and precipitates, leading to an increase in precipitates on the grain boundary and the widest non-precipitation zone. As shown in Figure 17b, it demonstrated the least corrosion resistance.

## 4. Conclusions

This study took the 2024-O aluminum alloy as the research object; the effects of high-temperature deformation on the mechanical properties and corrosion resistance of 2024-O aluminum alloy joints after FSW were investigated. The following conclusions can be drawn:

1. When there was no high-temperature deformation (0%), some coarse reinforcement particles were distributed in the matrix, and the mean value of reinforcement elements was 1.34 μm. The coarse particles were broken and the fine particles were evenly distributed after high-temperature deformation. When the high-temperature deformation was 20%, the mean value of reinforcement elements was only 1.19 μm.
2. In the welding state, the microhardness of the 2024-O aluminum alloy FSW joints showed an obvious “Ω” shape. The average microhardness of the weld nugget zone was the highest and its value was 111 HV. With the increase in high-temperature deformations, the overall hardness of the joints increased. When the high-temperature deformation was 20%, the microhardness and tensile strength of the FSW joint reached the maximum values of 146 HV and 465 MPa, respectively.
3. The FSW joints with high-temperature deformation of 0% and 20% had cleavage and dimple fractures. However, dimple fracture was the main fracture mode in the FSW joints with 10% and 30% deformation. Compared with the 0% deformation specimen, the hardness and strength values of the 20% deformation samples were increased by 32% and 21%, respectively. The corrosion resistance of the specimens was as follows from high to low: 20% > 0% > 30% > 10%.
4. Among the four deformed samples, the 20% deformation sample had the largest amount of uniformly distributed S’ phase, and the S’ phase’s shape was a very fine needle structure. This was mainly because the dislocation density increased continuously, which provided a more favorable nucleation position for the precipitation of the S’ phase.

## Data Availability

The data presented in this study are available on request from the corresponding author. The data are not publicly available due to privacy.
